# Adherence to Healthy and Unhealthy Plant-Based Diets and the Risk of Gout

**DOI:** 10.1001/jamanetworkopen.2024.11707

**Published:** 2024-05-21

**Authors:** Sharan K. Rai, Siyue Wang, Yang Hu, Frank B. Hu, Molin Wang, Hyon K. Choi, Qi Sun

**Affiliations:** 1 Department of Nutrition, Harvard T.H. Chan School of Public Health, Boston, Massachusetts; 2 Program in Population Health Sciences, Harvard Kenneth C. Griffin Graduate School of Arts and Sciences, Cambridge, Massachusetts; 3 Department of Epidemiology, Harvard T.H. Chan School of Public Health, Boston, Massachusetts; 4 Channing Division of Network Medicine, Department of Medicine, Brigham and Women’s Hospital and Harvard Medical School, Boston, Massachusetts; 5 Department of Biostatistics, Harvard T.H. Chan School of Public Health, Boston, Massachusetts; 6 Division of Rheumatology, Allergy, and Immunology, Massachusetts General Hospital and Harvard Medical School, Boston

## Abstract

**Importance:**

Plant-based diets are increasing in popularity due, in part, to their health benefits for selected cardiometabolic diseases as well as favorable environmental impact. Little is known about how such a diet is related to gout risk.

**Objective:**

To examine associations between adherence to a plant-based diet (including healthy and unhealthy versions of this diet), as well as its 18 individual food groups, and incident gout.

**Design, Setting, and Participants:**

This prospective cohort study used data from population-based cohorts of US men and women enrolled in the Health Professionals Follow-Up Study (1986-2012) and Nurses’ Health Study (1984-2010). Participants were men and women free of gout at baseline. Statistical analyses were performed over March 2020 to August 2023.

**Exposures:**

An overall plant-based diet index (PDI), as well as healthy (hPDI) and unhealthy (uPDI) versions of this index that emphasize healthy and less healthy plant-based foods, respectively. These diet indices were comprised of 18 food groups, assessed using a validated semiquantitative food frequency questionnaire.

**Main Outcomes and Measures:**

Incident cases of gout that were confirmed with a supplementary questionnaire to meet the preliminary American College of Rheumatology survey criteria for gout. Cox proportional hazards regression models were used to evaluate multivariable-adjusted associations of all 3 PDIs with incident gout using quintiles (Q) of adherence.

**Results:**

Among a total of 122 679 participants (mean [SD] age, 53.8 [9.8] years among 43 703 men; mean [SD] age, 50.9 [7.2] years among 78 976 women) over 2 704 899 person-years of follow-up, 2709 participants experienced incident gout. The overall PDI was not significantly associated with gout in either cohort (Q5 vs Q1 pooled hazard ratio [HR], 1.02 [95% CI, 0.89-1.17]; *P* for trend = .63). In the pooled analysis, hPDI was significantly inversely associated with risk of gout (Q5 vs Q1 HR, 0.79 [95% CI, 0.69-0.91]; *P* for trend = .002), while the uPDI was positively associated with risk of gout (Q5 vs Q1 HR, 1.17 [95% CI, 1.03-1.33]; *P* for trend = .02), particularly in women (Q5 vs Q1 HR, 1.31 [95% CI, 1.05-1.62]; *P* for trend = .01). In analysis of individual food groups, higher intakes of certain healthy plant foods, such as whole grains (pooled HR per 1 serving/d, 0.93 [95% CI, 0.89-0.97]) and tea and coffee (pooled HR per 1 serving/d, 0.95 [95% CI, 0.92-0.97]), as well as dairy (pooled HR per 1 serving/d, 0.86 [95% CI, 0.82-0.90]), were independently associated with a lower risk of gout, while selected unhealthy plant foods, such as fruit juice (pooled HR per 1 serving/d, 1.06 [95% CI, 1.00-1.13]) and sugar-sweetened beverages (pooled HR per 1 serving/d, 1.16 [95% CI, 1.07-1.26]) were associated with increased risk of gout.

**Conclusions and Relevance:**

The findings of this prospective cohort study of PDIs and gout support current dietary recommendations to increase consumption of healthy plant foods while lowering intake of unhealthy plant foods to mitigate gout risk.

## Key Points

**Question:**

Are healthy and unhealthy plant-based diets associated with the risk of developing gout?

**Findings:**

In this cohort study of 122 679 US men and women, adherence to an overall plant-based dietary pattern that includes both healthy and unhealthy plant foods was not associated with gout. However, higher intake of a healthy plant-based diet that specifically emphasizes healthier plant-based foods was associated with lower gout risk, while an unhealthy plant-based diet was associated with higher gout risk, particularly in women.

**Meaning:**

These findings support current dietary recommendations to increase consumption of healthy plant foods while lowering intake of less healthy plant foods to mitigate gout risk.

## Teaser

This cohort study assesses the associations of adherence to healthy and unhealthy plant-based diets with risk of gout in 2 large US cohorts.

## Introduction

Gout is the most common inflammatory arthritis, affecting approximately 4% of US adults,^1,2^ and is associated with an increased comorbidity burden, premature mortality, and negative mental health outcomes.^3,4,5,6^ Several dietary factors are associated with the risk of gout. Alcohol,^7^ red meat,^8^ fish,^8^ and sugar-sweetened beverages (SSBs)^9^ are all positively associated with gout, while low-fat dairy products,^8^ coffee,^10^ and vitamin C^11^ have shown inverse associations. Moreover, despite the moderate purine load of certain vegetables and legumes, they do not necessarily promote gout.^8^ While the contributions of selected individual food and beverage items (and nutrients) to gout risk are generally well characterized, these data are challenging to translate into dietary practices for gout prevention because foods are not eaten in isolation.^12^ Moreover, some foods that are positively associated with gout are simultaneously inversely associated with other cardiometabolic diseases (eg, fish).^13^ Therefore, it is not surprising that conventional purine-focused dietary advice for gout has confused patients.^14^

Dietary patterns better reflect how foods are consumed together, which may facilitate implementation of healthy eating patterns.^12^ The Dietary Approaches to Stop Hypertension (DASH) diet lowers serum urate,^15,16^ and is consistently inversely associated with gout in observational studies.^17,18^ A secondary analysis of the Dietary Intervention Randomized Controlled Trial found that a Mediterranean-style diet also lowered serum urate^19^ and was also inversely associated with gout.^18^ Although these dietary patterns share an emphasis on several healthy plant foods, plant-based diets that specifically deemphasize all animal products have received less attention. Such a diet is particularly timely, as the food system is inextricably linked with environmental sustainability^20^ and consuming a plant-based diet is a key measure to reduce growing environmental pressures as well as improve planetary health alongside human health.^21^ However, the limited prior studies of gout simply dichotomized plant-based diets as vegetarian vs meat-containing and did not differentiate between the quality of plant foods. For example, while SSBs are technically of plant origin, their nutrient profile is poor. Therefore, we examined the associations of an overall plant-based diet, as well as healthy and unhealthy versions of this diet, with the risk of incident gout in 2 large prospective cohorts of US men and women.

## Methods

The protocol for this cohort study was approved by the institutional review boards of Massachusetts General Hospital, Brigham and Women’s Hospital, and the Harvard T.H. Chan School of Public Health. Completion and return of study questionnaires implied informed consent. This study adheres to the Strengthening the Reporting of Observational Studies in Epidemiology (STROBE) reporting guideline for cohort studies.

### Study Population

The Nurses’ Health Study (NHS) began in 1976 with 121 700 female registered nurses (ages 30-55 years) and the Health Professionals Follow-Up Study (HPFS) began in 1986 with 51 529 male health professionals (ages 40-75 years). In our study, 1984 and 1986 were used as the baseline years for the NHS and HPFS, respectively, as these cycles are the first in which data on most covariates of interest were first collected. We excluded individuals who had prevalent gout, reported implausible energy intake (defined as <500 or >3500 kcal/d for women and <800 or >4200 kcal/d for men), or had incomplete dietary data at baseline. Our baseline study cohort consisted of 78 976 women in the NHS and 43 703 men in the HPFS.

### Dietary Assessment and the Plant-Based Indices

Detailed dietary information was collected every 4 years using a semiquantitative food frequency questionnaire. Participants provided information on their habitual consumption of approximately 130 food and beverage items over the previous year. Participants could select among 9 response categories that ranged from never or less than once per month to 6 or more times per day. The reliability and validity of the food frequency questionnaire have been discussed in detail elsewhere.^22,23,24,25^

We used 3 indices: an overall plant-based diet index (overall PDI), a healthy plant-based diet index (hPDI), and an unhealthy plant-based diet index (uPDI). The procedure used to develop these indices has been described in detail previously^26,27^ and is summarized in the eMethods in Supplement 1. Briefly, the overall PDI was constructed using 18 food groups that were further categorized as 1 of 7 healthy plant food groups, 5 unhealthy plant groups, or 6 animal food groups. All 3 PDIs could range from 18 to 90, with higher scores indicating greater adherence to the index. Higher scores across all 3 indices reflected lower animal food intake.

### Incident Gout Ascertainment

Participants who self-reported physician-diagnosed gout were mailed a supplementary questionnaire with the American College of Rheumatology survey criteria for gout to confirm the diagnosis.^28^ Incident gout was defined as meeting at least 6 of 11 survey criteria. A review was conducted by 2 board-certified rheumatologists for 50 men in the HPFS who self-reported a gout diagnosis, and the concordance rate between the survey criteria and medical records was 94%.^8^

### Covariate Assessment

Biennial questionnaires collected updated information on lifestyle factors, medical diagnoses, medication use, and current weight (response rate was approximately 90% in each cycle). The validity and reproducibility of self-reported body weight in these cohorts has been discussed previously.^29^ Briefly, self-reported weight was highly correlated (Pearson correlation coefficient = 0.97) with the mean of 2 standardized measurements taken by trained technicians 6 months apart.^29^ Participants in NHS also provided information on history of oral contraceptive use (baseline only), menopausal status, and hormone therapy use. Participants self-reported their race and ethnicity data, and race and ethnicity were categorized as Asian, Black, White, or other origin. For the NHS, other self-reported race and ethnicity included American Indian and Hawaiian. For the HPFS, other self-reported race and ethnicity included anyone who reported other origin in the questionnaire. We collected these data as part of their demographic information.

### Statistical Analysis

Person-time for each participant was calculated from the baseline questionnaire return date until either the date of gout diagnosis, death, date of last return of a valid questionnaire, or the end of the follow-up (HPFS: January 31, 2012; NHS: June 30, 2010). We divided the diet indices into quintiles (Qs) as this does not make any assumptions about linearity and limits the influence of outliers. We calculated cumulative mean values for all dietary variables to better reflect long-term diet and minimize within-person variation.^30^ We updated the values of nondietary variables every 2 years to account for any changes in these covariates over the follow-up. For body mass index (BMI) and physical activity, we attempted to minimize missing data by carrying forward valid observations from the previous cycle’s questionnaire for a single follow-up cycle, and we created missing indicators for any remaining missing values.

We used Cox proportional hazards regression models to evaluate the associations between each diet index (by Q) and incident gout. We used age (years) as the time scale with stratification by calendar time in 2-year intervals.^31,32^ The proportional hazards assumption was evaluated by including an interaction term between each Q of the diet indices and age. We did not detect any evidence of a violation of this assumption (*P* > .05 for all tests). Our multivariable models were adjusted for total energy intake, BMI, history of hypertension, history of kidney failure (HPFS only), diuretic use, physical activity, and alcohol intake. Among participants in NHS, we further adjusted for oral contraceptive use (baseline only), menopausal status, and hormone therapy use. All covariates were selected a priori for inclusion into our models based on whether they had the properties of a potential confounder in existing gout literature. We examined the *P* for trend by modeling the median values of the Qs as a continuous variable. We further examined potential nonlinear associations using restricted cubic splines with 4 knots, which was selected based on a comparison of the Akaike information criterion for each model.^33^ Tests for nonlinearity used the likelihood ratio test, comparing the model with only the linear term to the model with the linear and the cubic spline terms. In a sensitivity analysis, we imposed a 4-year lag period between diet assessment and gout incidence to evaluate the impact of potential reverse causation arising from preclinical manifestations of gout that may change diet. Finally, we examined the association of each of the 18 food groups with gout per additional daily serving.

Finally, we stratified our analyses of the hPDI and uPDI by hypertension status, overweight, dairy intake, total fiber intake, physical activity level, and vitamin C intake. These variables were chosen based on their public health and clinical significance. For instance, hypertension is a common comorbidity of gout, and dairy intake (an animal product that was reverse scored in the PDIs) has shown inverse associations with gout in prior research. We tested for interaction with each of these factors by including cross-product terms in our models and evaluating the *P* value for the interaction.

We analyzed both cohorts separately and pooled their hazard ratios (HRs) using a fixed-effects model. We used the Cochran *Q* statistic to assess potential heterogeneity across the cohorts. All statistical tests were 2-sided with a significance level of *P* = .05. We conducted all analyses using SAS software version 9.4 for UNIX (SAS Institute). We performed all statistical analyses between March 2020 and August 2023.

## Results

### Baseline Characteristics

A total of 122 679 participants (mean [SD] age, 53.8 [9.8] years among 43 703 men; mean [SD] age, 50.9 [7.2] years among 78 976 women) were assessed. Participants with higher PDI and hPDI scores tended to be older, more physically active, and consumed more total vitamin C (Table 1). Participants with higher uPDI scores were younger, less active, and consumed less vitamin C. Participants with higher scores for all 3 indices tended to have lower alcohol intake (Table 1). A full description of participant characteristics according to quintiles of the PDI, hPDI, and uPDI is shown in Table 1.

**Table 1.** Age-Standardized Baseline Characteristics of Men in the HPFS and Women in the NHS According to Quintile of Plant-Based Diet Indices

Characteristic	Mean (SD)^a^					
	HPFS (1986)			NHS (1984)		
	Q1	Q3	Q5	Q1	Q3	Q5
**Overall PDI**						
No. of participants	9459	10 453	8057	15 048	14 181	16 368
Overall PDI adherence	45.8 (3.0)	54.5 (1.1)	64.0 (2.9)	44.1 (2.9)	53.0 (0.8)	62.0 (2.9)
Age, y	53.1 (9.7)	53.7 (9.8)	54.5 (9.8)	50.3 (7.0)	50.8 (7.2)	51.7 (7.3)
Race and ethnicity, No. (%)						
Asian	146 (1.5)	183 (1.7)	120 (1.5)	86 (0.6)	91 (0.6)	144 (0.9)
Black	100 (1.1)	94 (0.9)	62 (0.8)	237 (1.6)	197 (1.4)	180 (1.1)
White	8989 (95.0)	9921 (94.9)	7698 (95.5)	14 688 (97.6)	13 859 (97.7)	16 001 (97.8)
Other	223 (2.4)	255 (2.4)	177 (2.2)	36 (0.2)	34 (0.2)	43 (0.3)
Physical activity, MET-h/wk	17.9 (22.8)	20.2 (23.7)	25.1 (27.9)	12.6 (19.0)	14.1 (22.1)	15.7 (22.9)
Body mass index	25.8 (3.4)	25.4 (3.1)	24.9 (3.0)	25.5 (5.1)	25.0 (4.6)	24.4 (4.3)
History of hypertension, No. %	1934 (20.4)	2068 (19.8)	1592 (19.8)	1410 (9.4)	1104 (7.8)	1178 (7.2)
History of kidney failure, No. (%)	13 (0.1)	14 (0.1)	3 (<0.1)	NA	NA	NA
Diuretic use, No. (%)	909 (9.6)	983 (9.4)	692 (8.6)	2066 (13.7)	1730 (12.2)	1782 (10.9)
Postmenopausal, No. (%)	NA	NA	NA	7071 (48.3)	6677 (48.2)	7723 (48.4)
Oral contraceptive use, No. (%)	NA	NA	NA	7441 (49.4)	6901 (48.7)	7774 (47.5)
Total energy intake, kcal/d	1689 (528)	1971 (584)	2339 (622)	1464 (456)	1717 (491)	2047 (520)
Alcohol consumption, g/d	12.7 (17.1)	10.9 (15.0)	9.7 (13.0)	8.6 (13.6)	6.7 (10.9)	5.9 (9.3)
Total vitamin C intake, mg/d	410 (517)	425 (469)	470 (442)	336 (438)	335 (385)	344 (333)
Food groups, servings/d						
Whole grains	1.0 (1.1)	1.5 (1.3)	2.3 (1.7)	0.7 (0.9)	1.0 (1.0)	1.5 (1.2)
Fruits	1.0 (0.9)	1.6 (1.2)	2.3 (1.6)	0.9 (0.8)	1.4 (1.0)	1.9 (1.1)
Vegetables	2.1 (1.3)	2.8 (1.6)	4.0 (2.1)	2.2 (1.3)	2.9 (1.5)	3.7 (1.8)
Nuts	0.2 (0.4)	0.4 (0.5)	0.6 (0.6)	0.1 (0.2)	0.2 (0.3)	0.3 (0.4)
Legumes	0.3 (0.2)	0.4 (0.3)	0.6 (0.4)	0.1 (0.2)	0.2 (0.2)	0.3 (0.2)
Vegetable oil	0.1 (0.3)	0.2 (0.3)	0.4 (0.5)	0.2 (0.3)	0.3 (0.4)	0.4 (0.4)
Tea and coffee	2.0 (1.9)	2.4 (1.9)	2.7 (2.0)	2.6 (1.9)	3.1 (1.9)	3.6 (2.0)
Fruit juice	0.5 (0.7)	0.8 (0.8)	1.1 (1.0)	0.4 (0.6)	0.7 (0.7)	1.0 (0.8)
Refined grains	1.1 (1.0)	1.5 (1.2)	1.9 (1.4)	1.3 (1.1)	1.7 (1.3)	2.3 (1.5)
Potatoes	0.4 (0.4)	0.6 (0.4)	0.7 (0.5)	0.4 (0.3)	0.5 (0.4)	0.7 (0.4)
Sugar-sweetened beverages	0.2 (0.6)	0.3 (0.6)	0.4 (0.6)	0.2 (0.6)	0.3 (0.6)	0.4 (0.6)
Sweets and desserts	0.9 (1.1)	1.4 (1.4)	1.9 (1.6)	0.8 (0.9)	1.2 (1.2)	1.8 (1.4)
Animal fat	0.4 (0.7)	0.3 (0.7)	0.1 (0.5)	0.5 (0.9)	0.4 (0.8)	0.3 (0.6)
Dairy	2.1 (1.6)	2.0 (1.4)	1.8 (1.3)	2.0 (1.4)	2.0 (1.4)	2.0 (1.3)
Eggs	0.4 (0.5)	0.3 (0.4)	0.2 (0.3)	0.4 (0.4)	0.3 (0.3)	0.3 (0.3)
Fish	0.4 (0.3)	0.4 (0.3)	0.4 (0.4)	0.3 (0.3)	0.3 (0.3)	0.3 (0.2)
Total meat	1.8 (0.9)	1.8 (0.9)	1.7 (1.0)	1.7 (0.8)	1.6 (0.8)	1.7 (0.8)
Miscellaneous animal foods	0.4 (0.4)	0.4 (0.4)	0.3 (0.3)	0.5 (0.4)	0.4 (0.4)	0.4 (0.4)
**hPDI**						
No. of participants	9188	8859	8283	16 850	16 402	16 998
hPDI adherence	44.6 (3.1)	54.5 (1.1)	65.6 (3.3)	43.5 (3.2)	53.5 (1.1)	63.6 (3.4)
Age, y	51.3 (9.4)	53.8 (9.7)	56.2 (9.5)	48.8 (7.1)	51.0 (7.1)	52.9 (6.8)
Race and ethnicity, No. (%)						
Asian	92 (1.0)	159 (1.8)	164 (2.0)	65 (0.4)	130 (0.8)	143 (0.8)
Black	76 (0.8)	82 (0.9)	70 (0.8)	191 (1.1)	218 (1.3)	241 (1.4)
White	8818 (96.0)	8430 (95.2)	7835 (94.6)	16 567 (98.3)	16 009 (97.6)	16 559 (97.4)
Other	202 (2.2)	188 (2.1)	214 (2.6)	27 (0.2)	45 (0.3)	54 (0.3)
Physical activity, MET-h/wk	17.8 (22.5)	19.9 (23.6)	25.9 (28.4)	11.1 (16.6)	13.5 (19.8)	18.1 (26.4)
Body mass index, kg/m^2^	25.5 (3.3)	25.5 (3.1)	25.0 (3.1)	25.4 (5.1)	25.0 (4.6)	24.5 (4.2)
History of hypertension, No. (%)	1678 (18.3)	1765 (19.9)	1738 (21.0)	1452 (8.6)	1246 (7.6)	1345 (7.9)
History of kidney failure, No. (%)	9 (0.1)	8 (0.1)	8 (0.1)	NA	NA	NA
Diuretic use, No. (%)	816 (8.9)	852 (9.6)	760 (9.2)	2088 (12.4)	1944 (11.9)	2105 (12.4)
Postmenopausal, No. (%)	NA	NA	NA	7874 (47.6)	7729 (48.4)	8070 (48.9)
Oral contraceptive use, No. (%)	NA	NA	NA	7995 (47.4)	7902 (48.2)	8396 (49.4)
Total energy intake, kcal/d	2343 (611)	1949 (580)	1706 (516)	2083 (504)	1712 (490)	1449 (440)
Alcohol consumption, g/d	11.4 (15.7)	11.4 (15.1)	10.4 (14.5)	6.9 (11.4)	7.0 (11.4)	6.6 (10.9)
Total vitamin C intake, mg/d	323 (353)	416 (459)	579 (582)	260 (283)	322 (360)	446 (484)
Food groups, servings/d						
Whole grains	1.1 (1.1)	1.5 (1.4)	2.2 (1.7)	0.7 (0.8)	1.1 (1.1)	1.4 (1.3)
Fruits	1.1 (0.9)	1.5 (1.2)	2.3 (1.7)	1.1 (0.8)	1.4 (1.0)	1.8 (1.2)
Vegetables	2.4 (1.3)	2.8 (1.7)	3.8 (2.3)	2.5 (1.3)	2.9 (1.5)	3.5 (1.9)
Nuts	0.3 (0.5)	0.4 (0.5)	0.4 (0.6)	0.2 (0.3)	0.2 (0.3)	0.3 (0.4)
Legumes	0.4 (0.3)	0.4 (0.3)	0.6 (0.4)	0.2 (0.1)	0.2 (0.2)	0.3 (0.3)
Vegetable oil	0.2 (0.3)	0.2 (0.3)	0.3 (0.5)	0.2 (0.3)	0.3 (0.3)	0.4 (0.5)
Tea and coffee	2.1 (1.9)	2.4 (1.9)	2.5 (2.0)	2.7 (1.8)	3.1 (2.0)	3.4 (2.0)
Fruit juice	0.9 (0.9)	0.8 (0.9)	0.6 (0.8)	0.9 (0.8)	0.7 (0.7)	0.5 (0.7)
Refined grains	2.2 (1.4)	1.4 (1.1)	1.0 (0.9)	2.5 (1.5)	1.7 (1.3)	1.1 (1.0)
Potatoes	0.8 (0.5)	0.5 (0.4)	0.4 (0.3)	0.7 (0.4)	0.5 (0.3)	0.3 (0.3)
Sugar-sweetened beverages	0.6 (0.8)	0.3 (0.5)	0.1 (0.3)	0.6 (0.8)	0.3 (0.5)	0.1 (0.3)
Sweets and desserts	2.1 (1.6)	1.4 (1.4)	0.7 (0.9)	1.9 (1.4)	1.2 (1.2)	0.7 (0.8)
Animal fat	0.6 (0.9)	0.2 (0.6)	0.1 (0.3)	0.7 (1.1)	0.3 (0.7)	0.2 (0.5)
Dairy	2.5 (1.6)	1.9 (1.4)	1.5 (1.2)	2.4 (1.4)	2.0 (1.3)	1.7 (1.2)
Eggs	0.5 (0.5)	0.3 (0.4)	0.2 (0.3)	0.5 (0.3)	0.3 (0.3)	0.3 (0.3)
Fish	0.4 (0.3)	0.4 (0.3)	0.4 (0.4)	0.3 (0.2)	0.3 (0.3)	0.3 (0.3)
Total meat	2.3 (1.0)	1.8 (0.9)	1.2 (0.7)	2.0 (0.8)	1.6 (0.7)	1.3 (0.7)
Miscellaneous animal foods	0.5 (0.4)	0.4 (0.4)	0.2 (0.3)	0.6 (0.4)	0.4 (0.4)	0.3 (0.3)
**uPDI**						
No. of participants	8438	9593	9234	16 066	15 237	17 154
uPDI adherence	44.6 (3.2)	54.5 (1.1)	64.4 (3.2)	44.1 (3.5)	55.5 (1.1)	65.8 (3.4)
Age, y	55.0 (9.4)	53.8 (9.7)	52.4 (10.0)	51.9 (6.9)	50.9 (7.2)	49.8 (7.3)
Race and ethnicity, No. (%)						
Asian	86 (1.0)	138 (1.4)	233 (2.5)	98 (0.6)	89 (0.6)	133 (0.8)
Black	57 (0.7)	83 (0.9)	126 (1.4)	170 (1.1)	192 (1.3)	284 (1.7)
White	8115 (96.2)	9165 (95.5)	8627 (93.4)	15 745 (98.0)	14 951 (97.9)	16 704 (97.4)
Other^b^	180 (2.1)	207 (2.2)	248 (2.7)	53 (0.3)	41 (0.3)	32 (0.2)
Physical activity, MET-h/wk	24.5 (25.9)	21.0 (25.0)	17.4 (22.9)	18.1 (25.3)	13.9 (20.0)	10.7 (16.9)
Body mass index, kg/m^2^	25.6 (3.4)	25.4 (3.2)	25.2 (3.1)	25.3 (4.7)	24.9 (4.7)	24.8 (4.8)
History of hypertension, No. (%)	1628 (19.3)	1944 (20.3)	1889 (20.5)	1260 (7.8)	1202 (7.9)	1405 (8.2)
History of kidney failure, No. (%)	9 (0.1)	9 (0.1)	9 (0.1)	NA	NA	NA
Diuretic use, No. (%)	746 (8.8)	895 (9.3)	868 (9.4)	2132 (13.3)	1821 (11.9)	1971 (11.5)
Postmenopausal, No. (%)	NA	NA	NA	7609 (48.6)	7229 (48.5)	8079 (48.3)
Oral contraceptive use, No. (%)	NA	NA	NA	8011 (49.9)	7387 (48.4)	8047 (46.9)
Total energy intake, kcal/d	2302 (620)	1966 (593)	1734 (545)	1969 (517)	1724 (513)	1536 (485)
Alcohol consumption, g/d	13.4 (16.2)	11.3 (15.0)	8.8 (13.9)	7.7 (11.6)	7.2 (11.5)	5.6 (10.6)
Total vitamin C intake, mg/d	527 (521)	427 (470)	358 (432)	413 (416)	329 (377)	276 (346)
Food groups, servings/d						
Whole grains	2.3 (1.6)	1.6 (1.5)	0.9 (1.0)	1.7 (1.3)	1.0 (1.0)	0.6 (0.7)
Fruits	2.3 (1.4)	1.6 (1.3)	1.0 (1.0)	2.1 (1.2)	1.3 (0.9)	0.8 (0.7)
Vegetables	4.3 (2.0)	2.9 (1.6)	1.8 (1.2)	4.3 (1.8)	2.8 (1.3)	1.8 (0.9)
Nuts	0.6 (0.7)	0.3 (0.5)	0.2 (0.4)	0.3 (0.4)	0.2 (0.3)	0.1 (0.2)
Legumes	0.6 (0.4)	0.4 (0.3)	0.3 (0.3)	0.3 (0.3)	0.2 (0.2)	0.1 (0.1)
Vegetable oil	0.4 (0.5)	0.2 (0.3)	0.1 (0.2)	0.5 (0.5)	0.3 (0.3)	0.1 (0.2)
Tea and coffee	3.0 (2.0)	2.4 (1.9)	1.7 (1.7)	3.7 (2.0)	3.1 (1.9)	2.5 (1.8)
Fruit juice	0.7 (0.9)	0.8 (0.8)	0.8 (0.9)	0.7 (0.8)	0.7 (0.7)	0.7 (0.8)
Refined grains	1.3 (1.1)	1.5 (1.2)	1.7 (1.3)	1.5 (1.3)	1.8 (1.4)	1.9 (1.4)
Potatoes	0.5 (0.4)	0.5 (0.4)	0.6 (0.5)	0.4 (0.3)	0.5 (0.4)	0.6 (0.4)
Sugar-sweetened beverages	0.2 (0.4)	0.3 (0.5)	0.5 (0.8)	0.1 (0.3)	0.3 (0.5)	0.5 (0.8)
Sweets and desserts	1.2 (1.2)	1.4 (1.4)	1.6 (1.5)	1.0 (1.1)	1.3 (1.2)	1.4 (1.3)
Animal fat	0.4 (0.8)	0.3 (0.6)	0.2 (0.5)	0.5 (0.8)	0.4 (0.8)	0.3 (0.7)
Dairy	2.5 (1.6)	1.9 (1.4)	1.5 (1.3)	2.6 (1.4)	1.9 (1.3)	1.4 (1.1)
Eggs	0.5 (0.5)	0.3 (0.4)	0.2 (0.4)	0.5 (0.4)	0.3 (0.3)	0.2 (0.3)
Fish	0.6 (0.4)	0.4 (0.3)	0.2 (0.2)	0.5 (0.3)	0.3 (0.2)	0.2 (0.1)
Total meat	2.1 (1.0)	1.7 (0.9)	1.5 (0.8)	1.9 (0.8)	1.6 (0.8)	1.4 (0.7)
Miscellaneous animal foods	0.5 (0.4)	0.4 (0.4)	0.3 (0.3)	0.6 (0.5)	0.4 (0.4)	0.3 (0.3)

Abbreviations: hPDI, healthy plant-based diet index; HPFS, Health Professionals Follow-Up Study; MET, metabolic equivalent task; NA, not applicable; NHS, Nurses’ Health Study; PDI, plant-based diet index; uPDI, unhealthy plant-based diet index.

^a^ Variables are standardized to the age distribution of the study population. Values of polytomous variables may not sum to 100% due to rounding.

^b^ For the NHS, other self-reported race and ethnicity included American Indian and Hawaiian. For the HPFS, other self-reported race included anyone who self-reported other origin.

### PDIs and Incident Gout

Over 2 704 899 person-years of follow-up, we documented 2709 participants with confirmed incident gout. After multivariable adjustment, the overall PDI was not significantly associated with gout in either cohort (Table 2). Comparing extreme quintiles, and after adjusting for all covariates, adherence to the overall PDI was not associated with incident gout (Q5 vs Q1 pooled HR, 1.02 [95% CI, 0.89-1.17]; *P* for trend = .63). In contrast, in the fully adjusted pooled analysis, adherence to the hPDI was significantly associated with decreased risk of gout (Q5 vs Q1 pooled HR, 0.79 [95% CI, 0.69-0.91]; *P* for trend = .002), and the association was more apparent in women (Q5 vs Q1 HR, 0.69 [95% CI 0.55-0.87]; *P* for trend < .001), while there was no significant association in men (Q5 vs Q1 HR, 0.85 [95% CI, 0.72-1.00]; *P* for trend = .12) (Table 2). Finally, in our fully adjusted models, uPDI was positively associated with risk of gout (Q5 vs Q1 pooled HR, 1.17 [95% CI, 1.03-1.33]; *P* for trend = .02), and similarly the positive association was more apparent in women (Q5 vs Q1 HR, 1.31 [95% CI, 1.05-1.62]; *P* for trend = .01) and not significant in men (Q5 vs Q1 HR, 1.10 [95% CI, 0.93-1.29]; *P* for trend = .32), although we did not observe statistically significant heterogeneity between cohorts (*P *for heterogeneity > .05). Adding a 4-year lag period between dietary assessments and gout incidence produced similar results (eTable 1 in Supplement 1).

**Table 2.** Cohort-Specific and Pooled Associations of Plant-Based Diet Indices With Gout Risk in the HPFS (1986-2012) and NHS (1984-2010) Cohorts

Measure	HR (95% CI)					HR (95% CI), per 10-unit increase	*P* value for trend^a^
	Q1	Q2	Q3	Q4	Q5		
**Overall PDI**							
HPFS							
Incident gout, events/PY	388/178 811	349/176 399	341/176 185	328/179 236	301/174 741	NA	NA
Age-adjusted model	1 [Reference]	0.91 (0.79-1.05)	0.88 (0.76-1.02)	0.84 (0.72-0.97)	0.79 (0.68-0.91)	0.87 (0.80-0.94)	.001
Multivariable model^b^	1 [Reference]	1.00 (0.86-1.16)	1.02 (0.88-1.18)	1.03 (0.88-1.20)	1.06 (0.90-1.26)	1.05 (0.95-1.15)	.42
NHS							
Incident gout, events/PY	244/361 575	225/360 976	190/363 590	185/371 291	158/362 095	NA	NA
Age-adjusted model	1 [Reference]	0.90 (0.75-1.08)	0.74 (0.61-0.89)	0.72 (0.59-0.87)	0.58 (0.47-0.71)	0.69 (0.61-0.78)	<.001
Multivariable model^b^	1 [Reference]	1.04 (0.87-1.26)	0.96 (0.79-1.17)	1.01 (0.82-1.25)	0.95 (0.75-1.20)	0.97 (0.84-1.12)	.72
Pooled (fixed effects)							
Age-adjusted model	1 [Reference]	0.91 (0.81-1.02)	0.83 (0.74-0.93)	0.79 (0.70-0.89)	0.70 (0.62-0.80)^c^	0.81 (0.76-0.87)^c^	<.001^c^
Multivariable model^b^	1 [Reference]	1.01 (0.90-1.14)	1.00 (0.88-1.12)	1.02 (0.90-1.16)	1.02 (0.89-1.17)	1.02 (0.95-1.11)	.63
**hPDI**							
HPFS							
Incident gout, events/PY	364/177 715	342/176 993	298/174 679	370/182 031	333/173 954	NA	NA
Age-adjusted model	1 [Reference]	0.92 (0.80-1.07)	0.81 (0.69-0.94)	0.95 (0.82-1.10)	0.88 (0.76-1.02)	0.95 (0.88-1.02)	.22
Multivariable model^b^	1 [Reference]	0.88 (0.76-1.02)	0.74 (0.63-0.87)	0.88 (0.75-1.02)	0.85 (0.72-1.00)	0.94 (0.87-1.02)	.12
NHS							
Incident gout, events/PY	215/361 931	202/361 635	234/370 466	192/358 224	159/367 271	NA	NA
Age-adjusted model	1 [Reference]	0.87 (0.72-1.06)	0.96 (0.79-1.15)	0.77 (0.63-0.94)	0.60 (0.49-0.74)	0.75 (0.68-0.83)	<.001
Multivariable model^b^	1 [Reference]	0.88 (0.72-1.07)	0.99 (0.81-1.20)	0.80 (0.64-0.98)	0.69 (0.55-0.87)	0.80 (0.71-0.90)	<.001
Pooled (fixed effects)							
Age-adjusted model	1 [Reference]	0.90 (0.80-1.02)	0.86 (0.77-0.97)	0.88 (0.79-0.99)	0.77 (0.69-0.87)^c^	0.88 (0.83-0.93)^c^	<.001^c^
Multivariable model^b^	1 [Reference]	0.88 (0.78-0.99)	0.83 (0.74-0.94)^c^	0.85 (0.75-0.96)	0.79 (0.69-0.91)	0.89 (0.83-0.95)^c^	.002
**uPDI**							
HPFS							
Incident gout, events/PY	355/177 578	351/176 537	339/175 928	334/177 984	328/177 345	NA	NA
Age-adjusted model	1 [Reference]	0.99 (0.86-1.15)	0.98 (0.85-1.14)	0.97 (0.83-1.12)	0.97 (0.83-1.13)	0.97 (0.90-1.04)	.63
Multivariable model^b^	1 [Reference]	1.03 (0.88-1.19)	1.03 (0.88-1.20)	1.02 (0.88-1.20)	1.10 (0.93-1.29)	1.04 (0.95-1.13)	.32
NHS							
Incident gout, events/PY	190/365 833	206/370 753	182/359 632	211/358 303	213/365 006	NA	NA
Age-adjusted model	1 [Reference]	1.10 (0.90-1.34)	0.98 (0.80-1.20)	1.15 (0.95-1.40)	1.18 (0.97-1.44)	1.10 (1.00-1.21)	.08
Multivariable model^b^	1 [Reference]	1.13 (0.92-1.38)	1.04 (0.84-1.28)	1.24 (1.01-1.53)	1.31 (1.05-1.62)	1.17 (1.05-1.30)	.01
Pooled (fixed effects)							
Age-adjusted model	1 [Reference]	1.03 (0.92-1.16)	0.98 (0.87-1.11)	1.03 (0.92-1.16)	1.04 (0.93-1.18)	1.01 (0.96-1.08)^c^	.49
Multivariable model^b^	1 [Reference]	1.06 (0.94-1.20)	1.03 (0.91-1.17)	1.10 (0.97-1.24)	1.17 (1.03-1.33)	1.08 (1.01-1.16)	.02

Abbreviations: hPDI, healthy plant-based diet index; HPFS, Health Professionals Follow-Up Study; HR, hazard ratio; NA, not applicable; NHS, Nurses’ Health Study; PDI, plant-based diet index; PY, person-years; uPDI, unhealthy plant-based diet index.

^a^ *P* value when we assigned the median value to each quintile and entered this as a continuous variable in the model.

^b^ Models were age and calendar time stratified and adjusted for energy intake (quintiles), body mass index (calculated as weight in kilograms divided by height in meters squared; <21, 21-22.9, 23-24.9, 25-26.9, 27-28.9, 29-30.9, 31-32.9, 33-34.9, ≥35, or missing), history of hypertension, history of kidney failure (men only), diuretic use, physical activity (quintiles or missing), and alcohol intake (0, 1-4, 5-9, 10-14, 15-29, or ≥30 g/d). Among women, the model was additionally adjusted for oral contraceptive use and menopausal status and postmenopausal hormone use (premenopausal, postmenopausal never, current, or past users, or missing).

^c^ *P* value for *Q*-statistic <.05, indicating statistically significant heterogeneity between cohorts.

**Figure 1.**
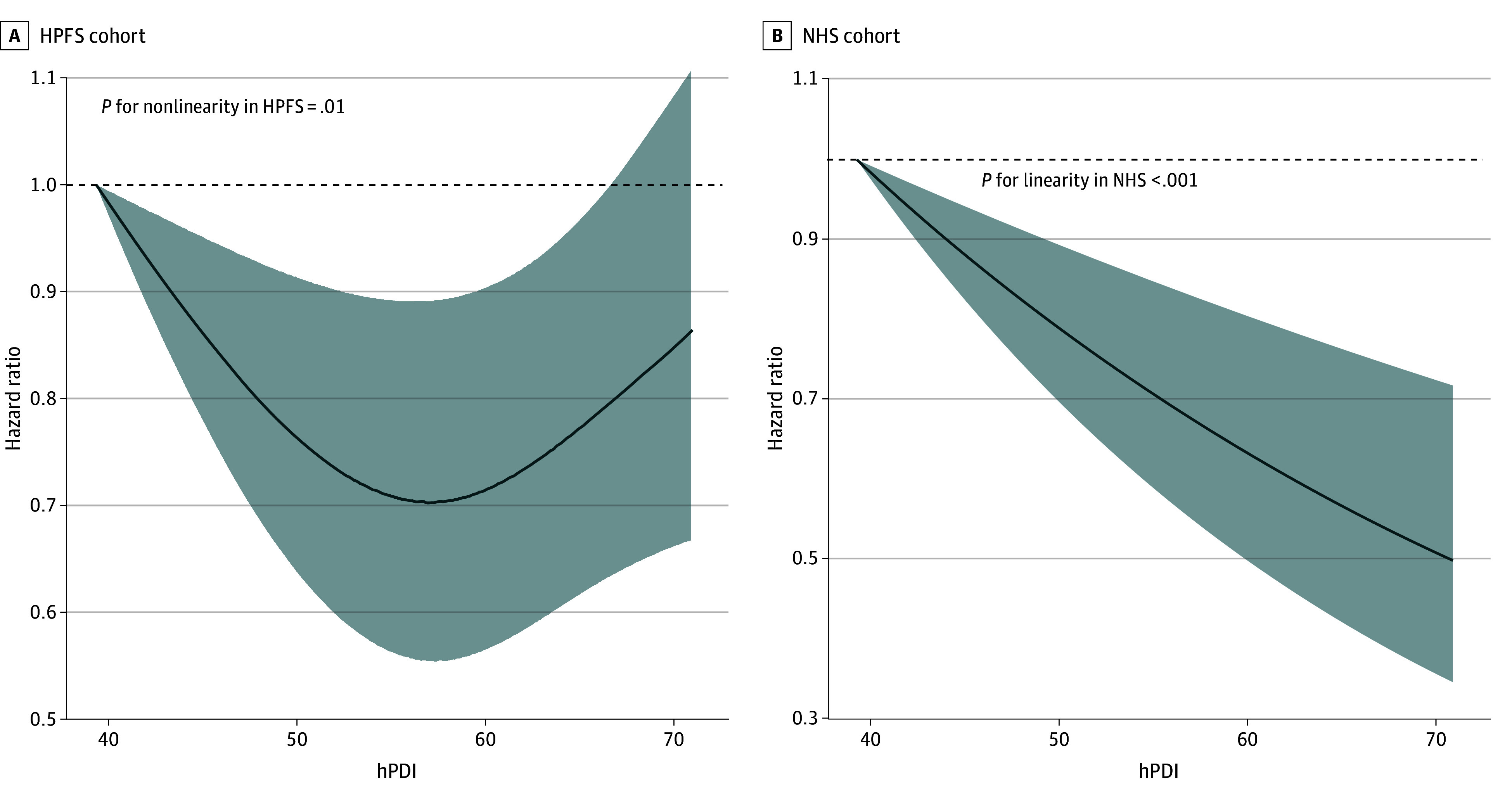
Dose-Response Associations of the Healthy Plant-Based Diet Index (hPDI) and Gout Incidence HPFS indicates Health Professionals Follow-Up Study; NHS, Nurses’ Health Study.

In our restricted cubic spline analysis, we found evidence for a nonlinear association between hPDI and gout among men. We observed a U-shaped association where gout risk was lowest between hPDI scores of approximately 55 to 60 (*P* for nonlinearity = .01) (Figure 1). We did not observe a similar deviation from linearity among women (*P* for linearity < .001).

### Food Groups and Incident Gout

**Figure 2.**
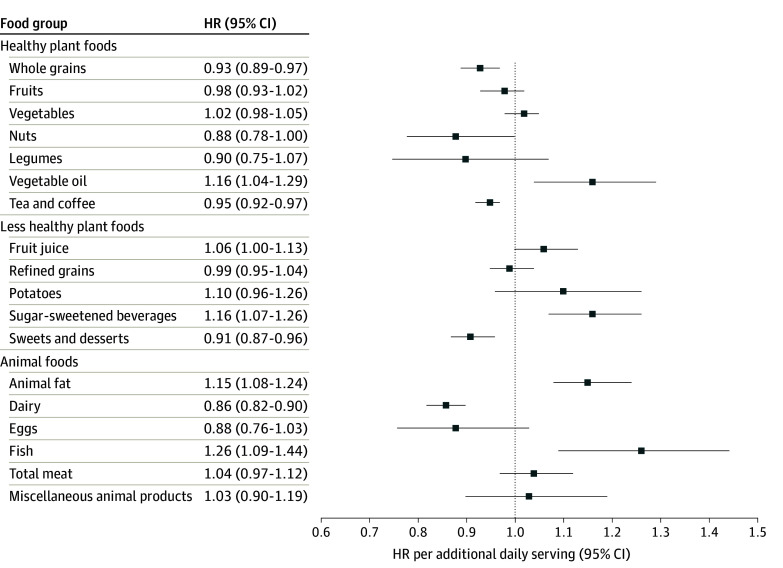
Pooled Associations of Each of the 18 Food Groups Comprising the Plant-Based Diet Indices and Incident Gout The model was age and calendar time stratified and adjusted for energy intake (quintiles), body mass index (calculated as weight in kilograms divided by height in meters squared; <21, 21-22.9, 23-24.9, 25-26.9, 27-28.9, 29-30.9, 31-32.9, 33-34.9, ≥35, or missing), history of hypertension, history of kidney failure (men only), diuretic use, physical activity (quintiles or missing), and alcohol intake (0, 1-4, 5-9, 10-14, 15-29, ≥30 g/d). Among women, the model was additionally adjusted for oral contraceptive use and menopausal status and postmenopausal hormone use (premenopausal, postmenopausal never, current, or past users, or missing). We also mutually adjusted for the other food groups in the same model.

Figure 2 shows the pooled associations between the intake of each of the 18 PDI food groups (for each serving/d increment) and incident gout. Among healthy plant foods, we found statistically significant inverse associations of whole grains (pooled HR per serving/d, 0.93 [95% CI, 0.89-0.97]) and tea or coffee (pooled HR per serving/d, 0.95 [95% CI, 0.92-0.97]; *P* for heterogeneity < .001) with risk of gout. We additionally found a significant association between vegetable oils and increased risk of gout (pooled HR per serving/d, 1.16 [95% CI, 1.04-1.29]). Fruits, vegetables, nuts, and legumes were not independently associated with gout. Among less healthy plant foods, fruit juices (pooled HR per serving/d, 1.06 [95% CI, 1.00-1.13]) and SSBs (pooled HR per serving/d, 1.16 [95% CI, 1.07-1.26]) were both associated with increased risk of gout, while sweets and desserts were associated with decreased risk (pooled HR per serving/d, 0.91 [95% CI, 0.87-0.96]). Neither refined grains nor potatoes were associated with gout. Finally, among animal foods, animal fats (pooled HR per serving/d, 1.15 [95% CI, 1.08-1.24]) and fish (pooled HR per serving/d, 1.26 [95% CI, 1.09-1.44]) were associated with increased risk of gout. Dairy intake was inversely associated with risk of gout (pooled HR per serving/d, 0.86 [95% CI, 0.82-0.90]), while eggs, total meat, and miscellaneous animal products were each not associated with gout. eTable 2 in Supplement 1 shows these associations by cohort.

### Subgroup Analysis

**Figure 3.**
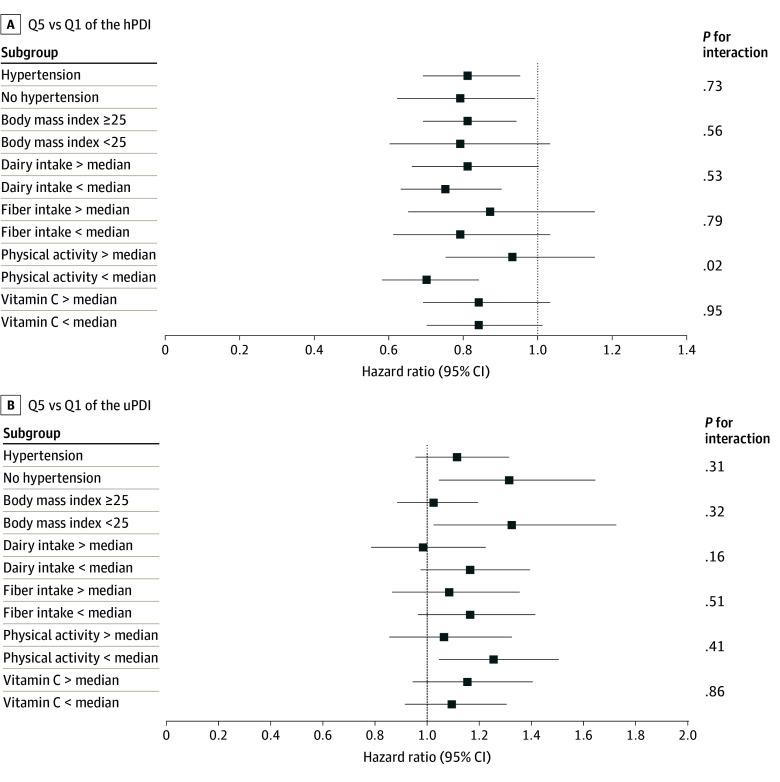
Pooled Hazard Ratios for Incident Gout Comparing Extreme Quintiles (Qs) of the Healthy Plant-Based Diet Index (hPDI) and Unhealthy Plant-Based Diet Index (uPDI) Stratified by Selected Characteristics All models were adjusted for the same covariates as in the main analysis except for the stratifying variables. Data are provided in eTable 3 in Supplement 1.

Similar associations were observed in strata defined by hypertension status, overweight, dairy intake, fiber intake, and vitamin C intake (Figure 3). For hPDI (not but uPDI), the inverse association with gout persisted among participants who were less physically active (Q5 vs Q1 pooled HR, 0.70 [95% CI, 0.58-0.84]) but was no longer significant among participants who were more physically active (Q5 vs Q1 pooled HR, 0.93 [95% CI, 0.75-1.15]) (*P* for interaction = .02). We found no other significant interactions.

## Discussion

In this large prospective cohort study of 122 679 men and women, we found that adherence to an overall plant-based dietary pattern that did not distinguish between healthy and less healthy plant foods was not associated with gout. However, higher intake of a healthy plant-based diet was associated with lower gout risk, while an unhealthy plant-based diet was associated with higher gout risk, particularly in women. These associations remained robust after adjustment for several potential confounders. We also found that higher intakes of certain food groups, such as whole grains, coffee and tea, dairy products, and, unexpectedly, sweets and desserts, were each independently inversely associated with incident gout, while other food groups, including fruit juices, SSBs, and fish, were positively associated with risk of gout.

While the DASH diet (which emphasizes fruits, vegetables, nuts, legumes, as well as dairy products) and the Mediterranean diet (which positively scores healthy plant foods as well as fish and moderate alcohol consumption) are associated with lower serum urate and gout risk,^15,16,17,18,19^ considerably less attention has been paid to diets that deemphasize all animal products. A study of 2 prospective Taiwan-based cohort studies found that lacto-ovo-vegetarians (ie, including dairy and/or egg products) had a lower risk of gout over 9 years.^34^ A cross-sectional analysis in the same study found that lacto-ovo-vegetarians had a lower serum urate level compared with both nonvegetarians and vegans (ie, individuals who consumed no dairy or eggs).^34^ A small study comparing Chinese Buddhist vegetarians with medical students found that vegetarian women, but not men, had lower serum urate levels than their omnivorous counterparts.^35^ A cross-sectional study using the EPIC-Oxford cohort found that lacto-ovo-vegetarians and fish-consumers both had lower serum urate than meat eaters and vegans, with vegans actually having the highest serum urate.^36^ Finally, another study reported that long-term (≥5 years) lacto-ovo-vegetarians had lower serum urate levels than age- and sex-matched nonvegetarian controls.^37^

Strict vegans or vegetarians are a small proportion of the population, particularly in the US (approximately 3% of individuals).^38^ In contrast, consuming plant-based diets that include small amounts of animal products has become increasingly popular,^38,39,40^ with the plant-based sector market recently valued at $7.4 billion dollars in the US.^41^ Therefore, it is important to understand how plant-based diets may affect gout risk, as this approach is likely more feasible at the population level. In our study, which is among the first studies examining PDIs, we further differentiated between a healthy vs less healthy PDI. Our findings provide the first prospective evidence, to our knowledge, that adherence to a healthy plant-based diet (ie, the hPDI), but not the overall PDI, was associated with a lower risk of gout, while a less healthy plant-based diet had the opposite association. The hPDI shares features with the DASH and Mediterranean diets, such as an emphasis on consuming fruits, vegetables, whole grains, nuts, and legumes. This adds to the growing literature of candidate dietary patterns for gout prevention with the additional benefit of promoting broader cardiometabolic and planetary health.

Regarding the associations for individual food groups, we found that higher intakes of dairy and tea or coffee were associated with lower gout risk, whereas higher intakes of fish, SSBs, and fruit juices were all associated with higher gout risk. These findings are in line with previous studies. Dairy has a well-established protective association for gout^8^ and can lower serum urate in short-term intervention studies (while eliminating dairy actually increases serum urate).^42,43^ Prior studies have found that coffee, but not tea, is inversely associated with serum urate and gout.^44,45^ SSBs and fruit juices, both high in fructose, were positively associated with gout in prior work.^9,46,47^ Fish, which is high in purines, is associated with higher gout risk.^8^ Interestingly, a study comparing individuals who consume fish (but not meat) with individuals who consume meat, vegetarians, and vegans found that those who ate fish had lower serum urate than vegans and those who ate meat.^36^ Women who consumed a Mediterranean-style diet (which positively scores fish intake) had a lower risk of gout.^18^ Finally, to our knowledge, this is the first study to show an inverse association between long-term intake of whole grain foods and gout.

We also made some unexpected observations. For example, we found a significant inverse association between sweets and gout, and a null association was observed for refined grains. In addition, we found that both animal fats and vegetable oils were associated with higher risk of gout. We also observed null associations between healthy fruits, vegetables, nuts, and legumes (as well as unhealthy potatoes) with incident gout, some of which are otherwise divergently associated with other metabolic conditions.^48,49^ Certain fruits high in fructose are positively associated with gout,^46^ which may explain the overall null finding when many fruits were examined collectively. A previous analysis of different purine-rich vegetables and legumes also did not identify any association with gout, which may be due to differences in the bioavailability of purines for metabolism to serum urate.^8^ Interestingly, we did not observe an association between total meat intake and gout, although prior work suggests that meat intake may be positively associated with both serum urate and incident gout.^50^ Collectively, the divergent pattern of associations for these individual food groups between gout and other cardiometabolic conditions suggest that the disease etiology relevant to these food groups may be different between gout and other metabolic conditions. Future studies ought to replicate these novel observations and work toward elucidating the potential underlying mechanisms. These unexpected findings also underscore that existing dietary patterns were developed for other, nongout conditions; therefore, their individual components may not be entirely consistent with gout risk factors (eg, dairy, which is of animal origin, was reverse scored in all 3 PDIs). Future studies to identify a tailored dietary pattern for gout prevention among individuals at high risk may be valuable.

There are likely several biological mechanisms underlying the associations of the PDIs with gout. The hPDI assessed in this study may help to prevent gout indirectly by improving insulin sensitivity. A recent mendelian randomization analysis supports the role of insulin resistance in inducing hyperuricemia, which is consistent with insulin’s known anti-uricosuric properties.^51^ The hPDI is also associated with less weight gain,^52^ which is a key risk factor for gout; however, we did adjust for BMI to evaluate a weight-independent pathway. Several foods and nutrients in the hPDI (or uPDI) may also contribute to a lower (or higher) risk of gout through independent pathways. Healthy plant-based diets tend to be rich in fiber which may help to resolve chronic inflammation,^53^ although prior studies of fiber intake and gout (or serum urate) are more limited. Vitamin C (found in citrus) is known to have a uricosuric effect and is associated with a lower risk of gout.^11,54^ Fructose, found in SSBs and fruit juices, can induce urate production through increased degradation of adenosine triphosphate to adenosine monophosphate.^55^ Chlorogenic acid (a polyphenol found in coffee) has weak xanthine oxidase activity and may lower serum urate.^56^ It remains unclear why we observed a nonlinear association of hPDI with gout among men but a linear association among women. This may reflect the effect of estrogen, which is known to play a role in gout onset (eg, postmenopausal women have a higher risk of gout compared with premenopausal women),^57^ and potential complex interactions between endogenous estrogen and certain plant-based compounds, such as phytoestrogens. This may be particularly relevant since phytoestrogens are metabolized by the gut microbiota^58^ and the microbiome has increasingly been shown to play a major role in gout pathogenesis.^59^

### Strengths and Limitations

The strengths and limitations of our study warrant discussion. To our knowledge, this is the first prospective evaluation of a plant-based diet (and plant food quality) in association with gout. Our study had a large sample size with confirmed incident gout cases, although we did not collect repeated measures of urate. We prospectively collected repeated measures of habitual food and beverage intake using a previously validated questionnaire before gout diagnosis, which minimizes potential recall bias. Measurement error remains a possibility, although this is not unique to our study. However, any measurement error is likely to be nondifferential with respect to our outcome and thus would likely bias our results toward the null. The use of repeated measures of diet and cumulative mean intake over the follow-up further minimizes random measurement error and within-participant variation. Our prospective study design and addition of a 4-year lag period in our sensitivity analysis allowed us to minimize the possibility of bias owing to reverse causation. Moreover, as urate is not routinely measured in primary care, it is unlikely that participants would modify their diet in response to reports of hyperuricemia. As with any observational study, we cannot rule out the possibility of residual confounding. It is also worth noting that the categorization of different plant foods into healthy vs less healthy groups is somewhat subjective. Furthermore, our study is comprised predominantly of White US-based health professionals, which may limit the generalizability of our findings to individuals belonging to similar racial and socioeconomic groups. However, it is worth noting that our study included a large population of women, which builds on earlier work that has disproportionately focused on men at risk of gout.

## Conclusions

This cohort study found evidence for an inverse association between a healthy plant-based dietary pattern and risk of incident gout, and a positive association between an unhealthy plant-based diet and risk of gout. Higher intakes of certain healthy plant foods, like whole grains and tea and coffee, as well as dairy, were independently associated with a lower risk of developing gout, while selected less healthy plant foods, like fruit juice and SSBs, were positively associated with gout. These findings support current dietary recommendations to increase consumption of healthy plant foods while lowering intake of less healthy plant foods to mitigate gout risk.

## Notes

Supplement 1.
eMethods.
**eTable 1.** Cohort-Specific and Pooled Associations Between Plant-Based Diet Indices and Gout Risk in the HPFS (1986-2012) and NHS (1984-2010) After Adding a 4-Year Lag Period Between Dietary Assessments and Gout Diagnosis **eTable 2.** Fully-Adjusted Hazard Ratios for Gout for Each Additional Serving of 18 Foods Groups in the HPFS (1986-2012) and NHS (1984-2010) Cohorts **eTable 3.** Pooled Hazard Ratio Data for Incident Gout Comparing Extreme Quintiles of the Healthy Plant-Based Diet Index (hPDI) and Unhealthy Plant-Based Diet Index (uPDI) Stratified by Selected Characteristics

Supplement 2.
Data Sharing Statement

## References

[1] Choi HK, Mount DB, Reginato AM; American College of Physicians; American Physiological Society. Pathogenesis of gout. Ann Intern Med. 2005;143(7):499-516. doi:10.7326/0003-4819-143-7-200510040-00009 16204163

[2] Chen-Xu M, Yokose C, Rai SK, Pillinger MH, Choi HK. Contemporary prevalence of gout and hyperuricemia in the United States and decadal trends: the National Health and Nutrition Examination Survey, 2007-2016. Arthritis Rheumatol. 2019;71(6):991-999. doi:10.1002/art.40807 30618180 PMC6536335

[3] Zhu Y, Pandya BJ, Choi HK. Comorbidities of gout and hyperuricemia in the US general population: NHANES 2007-2008. Am J Med. 2012;125(7):679-687.e1. doi:10.1016/j.amjmed.2011.09.033 22626509

[4] Fisher MC, Rai SK, Lu N, Zhang Y, Choi HK. The unclosing premature mortality gap in gout: a general population-based study. Ann Rheum Dis. 2017;76(7):1289-1294. doi:10.1136/annrheumdis-2016-210588 28122760

[5] Howren A, Bowie D, Choi HK, Rai SK, De Vera MA. Epidemiology of depression and anxiety in gout: a systematic review and metaanalysis. J Rheumatol. 2021;48(1):129-137. doi:10.3899/jrheum.190974 32115430

[6] Kleinstäuber M, Wolf L, Jones ASK, Dalbeth N, Petrie KJ. Internalized and anticipated stigmatization in patients with gout. ACR Open Rheumatol. 2020;2(1):11-17. doi:10.1002/acr2.11095 31943969 PMC6957912

[7] Choi HK, Atkinson K, Karlson EW, Willett W, Curhan G. Alcohol intake and risk of incident gout in men: a prospective study. Lancet. 2004;363(9417):1277-1281. doi:10.1016/S0140-6736(04)16000-5 15094272

[8] Choi HK, Atkinson K, Karlson EW, Willett W, Curhan G. Purine-rich foods, dairy and protein intake, and the risk of gout in men. N Engl J Med. 2004;350(11):1093-1103. doi:10.1056/NEJMoa035700 15014182

[9] Ebrahimpour-Koujan S, Saneei P, Larijani B, Esmaillzadeh A. Consumption of sugar sweetened beverages and dietary fructose in relation to risk of gout and hyperuricemia: a systematic review and meta-analysis. Crit Rev Food Sci Nutr. 2020;60(1):1-10. doi:10.1080/10408398.2018.1503155 30277800

[10] Park KY, Kim HJ, Ahn HS, . Effects of coffee consumption on serum uric acid: systematic review and meta-analysis. Semin Arthritis Rheum. 2016;45(5):580-586. doi:10.1016/j.semarthrit.2016.01.003 26905267

[11] Choi HK, Gao X, Curhan G. Vitamin C intake and the risk of gout in men: a prospective study. Arch Intern Med. 2009;169(5):502-507. doi:10.1001/archinternmed.2008.606 19273781 PMC2767211

[12] Cespedes EM, Hu FB. Dietary patterns: from nutritional epidemiologic analysis to national guidelines. Am J Clin Nutr. 2015;101(5):899-900. doi:10.3945/ajcn.115.110213 25832336 PMC4409695

[13] Zhang B, Xiong K, Cai J, Ma A. Fish consumption and coronary heart disease: a meta-analysis. Nutrients. 2020;12(8):2278. doi:10.3390/nu12082278 32751304 PMC7468748

[14] Rai SK, Choi HK, Choi SHJ, Townsend AF, Shojania K, De Vera MA. Key barriers to gout care: a systematic review and thematic synthesis of qualitative studies. Rheumatology (Oxford). 2018;57(7):1282-1292. doi:10.1093/rheumatology/kex530 29672772 PMC6014336

[15] Juraschek SP, Gelber AC, Choi HK, Appel LJ, Miller ER III. Effects of the Dietary Approaches to Stop Hypertension (DASH) diet and sodium intake on serum uric acid. Arthritis Rheumatol. 2016;68(12):3002-3009. doi:10.1002/art.39813 27523583 PMC5363397

[16] Juraschek SP, White K, Tang O, Yeh HC, Cooper LA, Miller ER III. Effects of a Dietary Approach to Stop Hypertension (DASH) diet intervention on serum uric acid in African Americans with hypertension. Arthritis Care Res (Hoboken). 2018;70(10):1509-1516. doi:10.1002/acr.23515 29342506 PMC6050157

[17] Rai SK, Fung TT, Lu N, Keller SF, Curhan GC, Choi HK. The Dietary Approaches to Stop Hypertension (DASH) diet, Western diet, and risk of gout in men: prospective cohort study. BMJ. 2017;357:j1794. doi:10.1136/bmj.j1794 28487277 PMC5423545

[18] Yokose C, McCormick N, Lu N, Joshi AD, Curhan G, Choi HK. Adherence to 2020 to 2025 Dietary Guidelines for Americans and the risk of new-onset female gout. JAMA Intern Med. 2022;182(3):254-264. doi:10.1001/jamainternmed.2021.7419 35099520 PMC8804972

[19] Yokose C, McCormick N, Rai SK, . Effects of low-fat, mediterranean, or low-carbohydrate weight loss diets on serum urate and cardiometabolic risk factors: a secondary analysis of the Dietary Intervention Randomized Controlled Trial (DIRECT). Diabetes Care. 2020;43(11):2812-2820. doi:10.2337/dc20-1002 33082244 PMC7576420

[20] Springmann M, Clark M, Mason-D’Croz D, . Options for keeping the food system within environmental limits. Nature. 2018;562(7728):519-525. doi:10.1038/s41586-018-0594-0 30305731

[21] Willett W, Rockström J, Loken B, . Food in the anthropocene: the EAT-Lancet Commission on healthy diets from sustainable food systems. Lancet. 2019;393(10170):447-492. doi:10.1016/S0140-6736(18)31788-4 30660336

[22] Rimm EB, Giovannucci EL, Stampfer MJ, Colditz GA, Litin LB, Willett WC. Reproducibility and validity of an expanded self-administered semiquantitative food frequency questionnaire among male health professionals. Am J Epidemiol. 1992;135(10):1114-1126. doi:10.1093/oxfordjournals.aje.a116211 1632423

[23] Willett WC, Sampson L, Stampfer MJ, . Reproducibility and validity of a semiquantitative food frequency questionnaire. Am J Epidemiol. 1985;122(1):51-65. doi:10.1093/oxfordjournals.aje.a114086 4014201

[24] Willett W. Nutritional Epidemiology. 3rd ed. Oxford University Press; 2013.

[25] Yuan C, Spiegelman D, Rimm EB, . Validity of a dietary questionnaire assessed by comparison with multiple weighed dietary records or 24-hour recalls. Am J Epidemiol. 2017;185(7):570-584. doi:10.1093/aje/kww104 28338828 PMC5859994

[26] Satija A, Bhupathiraju SN, Rimm EB, . Plant-based dietary patterns and incidence of type 2 diabetes in US men and women: results from three prospective cohort studies. PLoS Med. 2016;13(6):e1002039. doi:10.1371/journal.pmed.1002039 27299701 PMC4907448

[27] Satija A, Bhupathiraju SN, Spiegelman D, . Healthful and unhealthful plant-based diets and the risk of coronary heart disease in U.S. adults. J Am Coll Cardiol. 2017;70(4):411-422. doi:10.1016/j.jacc.2017.05.047 28728684 PMC5555375

[28] Wallace SL, Robinson H, Masi AT, Decker JL, McCarty DJ, Yü TF. Preliminary criteria for the classification of the acute arthritis of primary gout. Arthritis Rheum. 1977;20(3):895-900. doi:10.1002/art.1780200320 856219

[29] Rimm EB, Stampfer MJ, Colditz GA, Chute CG, Litin LB, Willett WC. Validity of self-reported waist and hip circumferences in men and women. Epidemiology. 1990;1(6):466-473. doi:10.1097/00001648-199011000-00009 2090285

[30] Hu FB, Stampfer MJ, Rimm E, . Dietary fat and coronary heart disease: a comparison of approaches for adjusting for total energy intake and modeling repeated dietary measurements. Am J Epidemiol. 1999;149(6):531-540. doi:10.1093/oxfordjournals.aje.a009849 10084242

[31] Korn EL, Graubard BI, Midthune D. Time-to-event analysis of longitudinal follow-up of a survey: choice of the time-scale. Am J Epidemiol. 1997;145(1):72-80. doi:10.1093/oxfordjournals.aje.a009034 8982025

[32] Breslow NE, Lubin JH, Marek P, Langholz B. Multiplicative Models and Cohort Analysis. J Am Stat Assoc. 1983;78:1-12. doi:10.1080/01621459.1983.10477915

[33] Durrleman S, Simon R. Flexible regression models with cubic splines. Stat Med. 1989;8(5):551-561. doi:10.1002/sim.4780080504 2657958

[34] Chiu THT, Liu CH, Chang CC, Lin MN, Lin CL. Vegetarian diet and risk of gout in two separate prospective cohort studies. Clin Nutr. 2020;39(3):837-844. doi:10.1016/j.clnu.2019.03.016 30955983

[35] Pan WH, Chin CJ, Sheu CT, Lee MH. Hemostatic factors and blood lipids in young Buddhist vegetarians and omnivores. Am J Clin Nutr. 1993;58(3):354-359. doi:10.1093/ajcn/58.3.354 8237846

[36] Schmidt JA, Crowe FL, Appleby PN, Key TJ, Travis RC. Serum uric acid concentrations in meat eaters, fish eaters, vegetarians and vegans: a cross-sectional analysis in the EPIC-Oxford cohort. PLoS One. 2013;8(2):e56339. doi:10.1371/journal.pone.0056339 23418557 PMC3572016

[37] Szeto YT, Kwok TC, Benzie IF. Effects of a long-term vegetarian diet on biomarkers of antioxidant status and cardiovascular disease risk. Nutrition. 2004;20(10):863-866. doi:10.1016/j.nut.2004.06.006 15474873

[38] Ruby MB. Vegetarianism: a blossoming field of study. Appetite. 2012;58(1):141-150. doi:10.1016/j.appet.2011.09.019 22001025

[39] Estell M, Hughes J, Grafenauer S. Plant protein and plant-based meat alternatives: consumer and nutrition professional attitudes and perceptions. Sustainability. 2021;13(3):1478. doi:10.3390/su13031478

[40] Alcorta A, Porta A, Tárrega A, Alvarez MD, Vaquero MP. Foods for plant-based diets: challenges and innovations. Foods. 2021;10(2):293. doi:10.3390/foods10020293 33535684 PMC7912826

[41] Nolden AA, Forde CG. The nutritional quality of plant-based foods. Sustainability. 2023;15(4):3324. doi:10.3390/su15043324

[42] Ghadirian P, Shatenstein B, Verdy M, Hamet P. The influence of dairy products on plasma uric acid in women. Eur J Epidemiol. 1995;11(3):275-281. doi:10.1007/BF01719431 7493659

[43] Garrel DR, Verdy M, PetitClerc C, Martin C, Brulé D, Hamet P. Milk- and soy-protein ingestion: acute effect on serum uric acid concentration. Am J Clin Nutr. 1991;53(3):665-669. doi:10.1093/ajcn/53.3.665 2000819

[44] Choi HK, Willett W, Curhan G. Coffee consumption and risk of incident gout in men: a prospective study. Arthritis Rheum. 2007;56(6):2049-2055. doi:10.1002/art.22712 17530645

[45] Choi HK, Curhan G. Coffee consumption and risk of incident gout in women: the Nurses’ Health Study. Am J Clin Nutr. 2010;92(4):922-927. doi:10.3945/ajcn.2010.29565 20739424 PMC2937590

[46] Choi HK, Curhan G. Soft drinks, fructose consumption, and the risk of gout in men: prospective cohort study. BMJ. 2008;336(7639):309-312. doi:10.1136/bmj.39449.819271.BE 18244959 PMC2234536

[47] Choi HK, Willett W, Curhan G. Fructose-rich beverages and risk of gout in women. JAMA. 2010;304(20):2270-2278. doi:10.1001/jama.2010.1638 21068145 PMC3058904

[48] Muraki I, Imamura F, Manson JE, . Fruit consumption and risk of type 2 diabetes: results from three prospective longitudinal cohort studies. BMJ. 2013;347:f5001. doi:10.1136/bmj.f5001 23990623 PMC3978819

[49] Afshin A, Micha R, Khatibzadeh S, Mozaffarian D. Consumption of nuts and legumes and risk of incident ischemic heart disease, stroke, and diabetes: a systematic review and meta-analysis. Am J Clin Nutr. 2014;100(1):278-288. doi:10.3945/ajcn.113.076901 24898241 PMC4144102

[50] Choi HK. A prescription for lifestyle change in patients with hyperuricemia and gout. Curr Opin Rheumatol. 2010;22(2):165-172. doi:10.1097/BOR.0b013e328335ef38 20035225

[51] McCormick N, O’Connor MJ, Yokose C, . Assessing the causal relationships between insulin resistance and hyperuricemia and gout using bidirectional mendelian randomization. Arthritis Rheumatol. 2021;73(11):2096-2104. doi:10.1002/art.41779 33982892 PMC8568618

[52] Satija A, Malik V, Rimm EB, Sacks F, Willett W, Hu FB. Changes in intake of plant-based diets and weight change: results from 3 prospective cohort studies. Am J Clin Nutr. 2019;110(3):574-582. doi:10.1093/ajcn/nqz049 31127828 PMC6735841

[53] Swann OG, Kilpatrick M, Breslin M, Oddy WH. Dietary fiber and its associations with depression and inflammation. Nutr Rev. 2020;78(5):394-411. doi:10.1093/nutrit/nuz072 31750916

[54] Brzezińska O, Styrzyński F, Makowska J, Walczak K. Role of vitamin C in prophylaxis and treatment of gout—a literature review. Nutrients. 2021;13(2):701. doi:10.3390/nu13020701 33671646 PMC7926958

[55] Caliceti C, Calabria D, Roda A, Cicero AFG. Fructose intake, serum uric acid, and cardiometabolic disorders: a critical review. Nutrients. 2017;9(4):395. doi:10.3390/nu9040395 28420204 PMC5409734

[56] Zhao M, Zhu D, Sun-Waterhouse D, . In vitro and in vivo studies on adlay-derived seed extracts: phenolic profiles, antioxidant activities, serum uric acid suppression, and xanthine oxidase inhibitory effects. J Agric Food Chem. 2014;62(31):7771-7778. doi:10.1021/jf501952e 25029106

[57] Hak AE, Curhan GC, Grodstein F, Choi HK. Menopause, postmenopausal hormone use and risk of incident gout. Ann Rheum Dis. 2010;69(7):1305-1309. doi:10.1136/ard.2009.109884 19592386 PMC3142742

[58] Rietjens IMCM, Louisse J, Beekmann K. The potential health effects of dietary phytoestrogens. Br J Pharmacol. 2017;174(11):1263-1280. doi:10.1111/bph.13622 27723080 PMC5429336

[59] Tong S, Zhang P, Cheng Q, . The role of gut microbiota in gout: is gut microbiota a potential target for gout treatment. Front Cell Infect Microbiol. 2022;12:1051682. doi:10.3389/fcimb.2022.1051682 36506033 PMC9730829

