# Supplementary material for: Adherence to Healthy and Unhealthy Plant-Based Diets and the Risk of Gout
Source: JAMA Netw Open. 2024 May 21;7(5):e2411707. doi: 10.1001/jamanetworkopen.2024.11707 (PMC11109774; doi:10.1001/jamanetworkopen.2024.11707)
Supplement: Supplement 1. — eMethods. eTable 1. Cohort-Specific and Pooled Associations Between Plant-Based Diet Indices and Gout Risk in the HPFS (1986-2012) and NHS (1984-2010) After Adding a 4-Year Lag Period Between Dietary Assessments and Gout Diagnosis eTable 2. Fully-Adjusted Hazard Ratios for Gout for Each Additional Serving of 18 Foods Groups in the HPFS (1986-2012) and NHS (1984-2010) Cohorts eTable 3. Pooled Hazard Ratio Data for Incident Gout Comparing Extreme Quintiles of the Healthy Plant-Based Diet Index (hPDI) and Unhealthy Plant-Based Diet Index (uPDI) Stratified by Selected Characteristics [file jamanetwopen-e2411707-s001.pdf]

Supplemental Online Content

Rai SK, Wang S, Hu Y, et al. Adherence to healthy and unhealthy plant-based diets and the risk of gout. *JAMA Netw Open*. 2024;7(5):e2411707.
doi:10.1001/jamanetworkopen.2024.11707

eMethods.
eTable 1. Cohort-Specific and Pooled Associations Between Plant-Based Diet Indices and Gout Risk in the HPFS (1986-2012) and NHS (1984-2010) After Adding a 4-Year Lag Period Between Dietary Assessments and Gout Diagnosis
eTable 2. Fully-Adjusted Hazard Ratios for Gout for Each Additional Serving of 18 Foods Groups in the HPFS (1986-2012) and NHS (1984-2010) Cohorts
eTable 3. Pooled Hazard Ratio Data for Incident Gout Comparing Extreme Quintiles of the Healthy Plant-Based Diet Index (hPDI) and Unhealthy Plant-Based Diet Index (uPDI) Stratified by Selected Characteristics

This supplemental material has been provided by the authors to give readers additional information about their work.

© 2024 Rai SK et al. *JAMA Network Open.*

eMethods

Development of the Plant-Based Diet Indices

To develop the plant-based diet indices, the consumption frequency of each food was converted into the number of daily servings. The number of servings of each food was then summed and assigned to one of 18 food groups based on their shared nutrient and culinary properties. Each food category could be further grouped into healthy plant foods, less healthy plant foods, and animal foods. Plant foods were classified as healthy or less healthy based on current evidence of their associations with several cardiometabolic outcomes including type 2 diabetes, cardiovascular disease, and intermediate conditions including obesity and hypertension. Alcohol was not included in the indices because it has an opposing direction of association for different health outcomes.

Each of the 18 food groups was divided into quintiles. For the PDI, participants were given a score of 1-5 for each plant food group (whether healthy or unhealthy) corresponding to their quintiles (positive scoring). This scoring was reversed for animal foods where participants received a score of 1 for each animal food group when they were at the highest quintile of consumption (i.e., reverse scoring). For the hPDI, positive scores were used for the healthy plant food groups, while reverse scores were given to unhealthy plant foods as well as animal foods. Conversely, for uPDI, positive scores were used for less healthy plant foods, while reverse scores were used for the healthy plant foods and animal foods.

© 2024 Rai SK et al. *JAMA Network Open.*

eTable 1. Cohort-specific and pooled associations between plant-based diet indices and gout risk in the HPFS (1986-2012) and NHS (1984-2010) after adding a 4-year lag period between dietary assessments and gout diagnosis

	Q1	Q2	Q3	Q4	Q5	Per 10-unit increase	P for trend‡
			**PDI**				
**HPFS**							
Main results	1.0 (Reference)	1.00 (0.86, 1.16)	1.02 (0.88, 1.18)	1.03 (0.88, 1.20)	1.06 (0.90, 1.26)	1.05 (0.95, 1.15)	0.42
Lagged analysis	1.0 (Reference)	1.00 (0.85, 1.17)	0.98 (0.83, 1.15)	1.02 (0.86, 1.20)	1.08 (0.90, 1.29)	1.05 (0.95, 1.17)	0.36
**NHS**							
Main results	1.0 (Reference)	1.04 (0.87, 1.26)	0.96 (0.79, 1.17)	1.01 (0.82, 1.25)	0.95 (0.75, 1.20)	0.97 (0.84, 1.12)	0.72
Lagged analysis	1.0 (Reference)	1.08 (0.89, 1.31)	0.99 (0.80, 1.21)	1.07 (0.86, 1.33)	1.01 (0.80, 1.28)	1.01 (0.87, 1.18)	0.86
**Pooled (fixed effects)**							
Main results	1.0 (Reference)	1.01 (0.90, 1.14)	1.00 (0.88, 1.12)	1.02 (0.90, 1.16)	1.02 (0.89, 1.17)	1.02 (0.95, 1.11)	0.63
Lagged analysis	1.0 (Reference)	1.03 (0.91, 1.16)	0.98 (0.86, 1.12)	1.04 (0.91, 1.18)	1.05 (0.91, 1.22)	1.04 (0.96, 1.13)	0.40
			**hPDI**				
**HPFS**							
Main results	1.0 (Reference)	0.88 (0.76, 1.02)	0.74 (0.63, 0.87)	0.88 (0.75, 1.02)	0.85 (0.72, 1.00)	0.94 (0.87, 1.02)	0.12
Lagged analysis	1.0 (Reference)	0.89 (0.76, 1.05)	0.75 (0.63, 0.89)	0.86 (0.73, 1.02)	0.86 (0.72, 1.02)	0.93 (0.85, 1.01)	0.11
**NHS**							
Main results	1.0 (Reference)	0.88 (0.72, 1.07)	0.99 (0.81, 1.20)	0.80 (0.64, 0.98)	0.69 (0.55, 0.87)	0.80 (0.71, 0.90)	0.0008
Lagged analysis	1.0 (Reference)	0.87 (0.71, 1.07)	1.00 (0.82, 1.23)	0.77 (0.62, 0.96)	0.71 (0.56, 0.90)	0.82 (0.72, 0.92)	0.002
**Pooled (fixed effects)**							
Main results	1.0 (Reference)	0.88 (0.78, 0.99)	0.83 (0.74, 0.94)†	0.85 (0.75, 0.96)	0.79 (0.69, 0.91)	0.89 (0.83, 0.95)†	0.002
Lagged analysis	1.0 (Reference)	0.89 (0.78, 1.00)	0.85 (0.74, 0.96)†	0.83 (0.73, 0.95)	0.80 (0.70, 0.92)	0.89 (0.83, 0.95)	0.002
			**uPDI**				
**HPFS**							
Main results	1.0 (Reference)	1.03 (0.88, 1.19)	1.03 (0.88, 1.20)	1.02 (0.88, 1.20)	1.10 (0.93, 1.29)	1.04 (0.95, 1.13)	0.32
Lagged analysis	1.0 (Reference)	1.08 (0.92, 1.26)	1.02 (0.87, 1.21)	1.03 (0.87, 1.22)	1.16 (0.97, 1.38)	1.05 (0.96, 1.15)	0.18
**NHS**							
Main results	1.0 (Reference)	1.13 (0.92, 1.38)	1.04 (0.84, 1.28)	1.24 (1.01, 1.53)	1.31 (1.05, 1.62)	1.17 (1.05, 1.30)	0.01
Lagged analysis	1.0 (Reference)	1.10 (0.90, 1.36)	1.08 (0.87, 1.34)	1.21 (0.98, 1.50)	1.27 (1.01, 1.58)	1.15 (1.03, 1.29)	0.03
**Pooled (fixed effects)**							
Main results	1.0 (Reference)	1.06 (0.94, 1.20)	1.03 (0.91, 1.17)	1.10 (0.97, 1.24)	1.17 (1.03, 1.33)	1.08 (1.01, 1.16)	0.02
Lagged analysis	1.0 (Reference)	1.09 (0.96, 1.23)	1.05 (0.92, 1.19)	1.10 (0.96, 1.25)	1.20 (1.04, 1.37)	1.09 (1.02, 1.17)	0.01

†P-value for *Q*-statistic <0.05, indicating statistically significant heterogeneity between the two cohorts.
‡P-value when we assigned the median value to each quintile and entered this as a continuous variable in the model.

© 2024 Rai SK et al. *JAMA Network Open.*

eTable 2. Fully-adjusted hazard ratios (HRs [95% CI]) for gout for each additional serving of 18 foods groups in the HPFS (1986-2012) and NHS (1984-2010)

	HPFS		NHS		Pooled (fixed effects)†	
**Food group**	**HR per additional daily serving**	**P for trend‡**	**HR per additional daily serving**	**P for trend‡**	**HR per additional daily serving**	**P for trend‡**
**Healthy plant foods**						
Whole grains	0.95 (0.90, 1.00)	0.04	0.87 (0.79, 0.96)	0.006	0.93 (0.89, 0.97)	0.002
Fruits	0.99 (0.94, 1.05)	0.76	0.94 (0.85, 1.03)	0.17	0.98 (0.93, 1.02)	0.34
Vegetables	1.00 (0.97, 1.04)	0.85	1.04 (0.98, 1.11)	0.16	1.02 (0.98, 1.05)	0.36
Nuts	0.86 (0.75, 0.99)	0.03	1.02 (0.75, 1.37)	0.92	0.88 (0.78, 1.00)	0.05
Legumes	0.86 (0.71, 1.04)	0.12	1.16 (0.73, 1.86)	0.53	0.90 (0.75, 1.07)	0.22
Vegetable oil	1.11 (0.97, 1.26)	0.12	1.27 (1.05, 1.54)	0.01	1.16 (1.04, 1.29)	0.008
Tea and coffee	0.99 (0.96, 1.02)	0.34	0.87 (0.83, 0.91)	<0.0001	0.95 (0.92, 0.97)†	<0.0001†
**Unhealthy plant foods**						
Fruit juice	1.05 (0.98, 1.12)	0.20	1.11 (0.99, 1.24)	0.07	1.06 (1.00, 1.13)	0.04
Refined grains	0.99 (0.94, 1.04)	0.65	1.00 (0.92, 1.08)	0.96	0.99 (0.95, 1.04)	0.68
Potatoes	1.07 (0.91, 1.25)	0.41	1.19 (0.92, 1.54)	0.19	1.10 (0.96, 1.26)	0.17
Sugar-sweetened beverages	1.15 (1.04, 1.27)	0.006	1.18 (1.02, 1.37)	0.03	1.16 (1.07, 1.26)	0.0004
Sweets and desserts	0.91 (0.86, 0.96)	0.001	0.92 (0.84, 1.01)	0.09	0.91 (0.87, 0.96)	0.0002
**Animal foods**						
Animal fat	1.20 (1.10, 1.31)	<0.0001	1.08 (0.97, 1.21)	0.17	1.15 (1.08, 1.24)	<0.0001
Dairy	0.85 (0.81, 0.90)	<0.0001	0.87 (0.81, 0.95)	0.001	0.86 (0.82, 0.90)	<0.0001
Eggs	0.83 (0.70, 0.99)	0.04	1.05 (0.78, 1.43)	0.74	0.88 (0.76, 1.03)	0.10
Fish	1.27 (1.09, 1.48)	0.002	1.19 (0.85, 1.65)	0.31	1.26 (1.09, 1.44)	0.001
Total meat	1.01 (0.93, 1.09)	0.87	1.14 (0.99, 1.31)	0.07	1.04 (0.97, 1.12)	0.29
Misc. animal products	0.98 (0.83, 1.17)	0.84	1.15 (0.89, 1.48)	0.29	1.03 (0.90, 1.19)	0.67

The model was age and calendar time stratified and adjusted for energy intake (quintiles), body mass index (<21, 21-22.9, 23-24.9, 25-26.9, 27-28.9, 29-30.9, 31-32.9, 33-34.9, ≥35 kg/m^2, missing), history of hypertension, history of renal failure (men only), diuretic use, physical activity (quintiles or missing), and alcohol intake (0, 1-4, 5-9, 10-14, 15-29, 30+ g/day). Among women, the model was additionally adjusted for oral contraceptive use and menopausal status/postmenopausal hormone use (premenopausal, postmenopausal never, current, or past users, or missing). We also mutually adjusted for the other food groups in the same model.

†P-value for *Q*-statistic <0.05, indicating statistically significant heterogeneity between the two cohorts.

‡P-value corresponding to the continuous variable.

© 2024 Rai SK et al. *JAMA Network Open.*

eTable 3. Pooled Hazard Ratio Data for Incident Gout Comparing Extreme Quintiles of the Healthy Plant-Based Diet Index (hPDI) and Unhealthy Plant-Based Diet Index (uPDI) Stratified by Selected Characteristics

Group	hPDI		uPDI	
	HR (95% CI)	P for interaction	HR (95% CI)	P for interaction
Hypertension	0.81 (0.69, 0.95)	0.73	1.11 (0.95, 1.31)	0.31
No hypertension	0.79 (0.62, 0.99)		1.31 (1.04, 1.64)	
BMI ≥ 25 kg/m^2	0.81 (0.69, 0.94)	0.56	1.02 (0.88, 1.19)	0.32
BMI < 25 kg/m^2	0.79 (0.60, 1.03)		1.32 (1.02, 1.72)	
Dairy intake > median	0.81 (0.66, 1.00)	0.53	0.98 (0.78, 1.22)	0.16
Dairy intake < median	0.75 (0.63, 0.90)		1.16 (0.97, 1.39)	
Fiber intake > median	0.87 (0.65, 1.15)	0.79	1.08 (0.86, 1.35)	0.51
Fiber intake < median	0.79 (0.61, 1.03)		1.16 (0.96, 1.41)	
Physical activity > median	0.93 (0.75, 1.15)	0.02	1.06 (0.85, 1.32)	0.41
Physical activity < median	0.70 (0.58, 0.84)		1.25 (1.04, 1.50)	
Vitamin C > median	0.84 (0.69, 1.03)	0.95	1.15 (0.94, 1.40)	0.86
Vitamin C < median	0.84 (0.70, 1.01)		1.09 (0.91, 1.30)	

All models were adjusted for the same covariates as in the main analysis except for the stratifying variables.

© 2024 Rai SK et al. *JAMA Network Open.*
