# Supplementary material for: Adherence to Healthy and Unhealthy Plant-Based Diets and the Risk of Gout
Source: JAMA Netw Open. 2024 May 21;7(5):e2411707. doi: 10.1001/jamanetworkopen.2024.11707 (PMC11109774; doi:10.1001/jamanetworkopen.2024.11707)
Supplement: Supplement 2. — Data Sharing Statement [file jamanetwopen-e2411707-s002.pdf]

Data Sharing Statement

Rai. Adherence to Healthy and Unhealthy Plant-Based Diets and the Risk of Gout. *JAMA Netw Open*. Published May 21, 2024. doi:10.1001/jamanetworkopen.2024.11707

Data
Data available: No
